# Supplementary material for: Low venous thromboembolism incidence in high risk medical patients in an Israeli hospital. Can risk assessment be extrapolated to different populations?
Source: PLoS One. 2020 Jul 6;15(7):e0235683. doi: 10.1371/journal.pone.0235683 (PMC7337280; doi:10.1371/journal.pone.0235683)
Supplement: Attachment — Submitted filename: response to reviewers 01.06.2020.docx [file pone.0235683.s002.docx]

Monday, June 1, 2020

**Dear PLOS ONE Editorial Office,**

First, I would like to thank the reviewers for their comprehensive and meticulous review and the PLOS ONE journal editors for their decision to reconsider the article for publication.

General comments:

1. The manuscript underwent extensive changes based on reviewer's comments. All changes have been highlighted in the corresponding file and labeled "revised manuscript with track changes". I will address to the specific points of the reviewers in the following paragraph. Additional unmarked version of the article will also be submitted.
2. The financial disclosure was not change
3. The manuscript has been formatted according PLOS ONE style. Tables were incorporated into text; figures were removed within the manuscript file and formatted according PACE digital diagnostics in separate files. Reference has been formatted.
4. Repository Data will be available upon acceptation. I do not have a DOI or website to upload the repository data. I will need your assistance with that.
5. The full name of the ethics committee was included in the Methods section of the manuscript and in the submission form.
6. The Authors’ affiliations were corrected to ensure that each author is linked to an affiliation.

I will address each point of the reviewers.

Reviewer #1:

Major comments

1. Identification and capture of VTE events - The main limitation of this study is its retrospective design. It is very surprising that there were no in-hospital diagnoses of pulmonary embolism among >700 medical inpatients. Reporting the number of chest CT and leg compression ultrasounds performed in this sample would be very important, given their routine use for in-hospital care. Also, the capture of VTE events after discharge is not convincing enough. Authors should provide more details on the Clalit Health Services database, and how confident they can be not to have missed VTE events in the retrospective follow-up. Exploring “unexpected” death remains quite vague, especially retrospectively, as fatal PE can occur in patients with co-morbidities and not be recognized. –
   a. “The main limitation of this study is its retrospective design” – We address this in the limitation paragraph. “This was a retrospective study which on one hand presents a disadvantage in design but on the other hand, works to our advantage since there was no need to obtain the patients signed consent to participate in the study as required in the previous studies (Padua and Geneva). Hence, our study included seriously ill patients at high risk of symptomatic VTE and VTE related death in which we would not have been able to obtain signed consent if a prospective design was adopted. This population probably reflects reality more accurately”.
   b. “It is very surprising that there were no in-hospital diagnoses of pulmonary embolism among >700 medical inpatients. Reporting the number of chest CT and leg compression ultrasounds performed in this sample would be very important, given their routine use for in-hospital care” – Our study, as opposed to others, didn’t search for asymptomatic VTE. The routine use of VTE diagnosis used in our hospital are Chest CTA for PE and US doppler for Distal DVT when suspected. Alternative modalities such as Transesophageal Echocardiography and Ventilation perfusion scan are used when C/I appear as per guidelines. We cannot provide information regarding patients who seek medical care or complain due to PE or DVT and discharge with a wrong diagnosis. It is unlikely that patients will not seek medical therapy again when PE/DVT was missed.
   c. “The capture of VTE events after discharge is not convincing enough. Authors should provide more details on the Clalit Health Services database, and how confident they can be not to have missed VTE events in the retrospective follow-up” We address this issue in the limitation section and updated the paragraph. “Clalit Health Services is the largest of the four health funds with around 3.8 million insured members, 54% of the Israeli population. The HMO's computer system constantly updating medical information from all medical services including other health service organizations and therefore the risk of missing vital information is significantly low. Furthermore, when we suspected that vital medical information was incomplete or missing, we contacted the patient directly”.
   d. Exploring “unexpected” death remains quite vague, especially retrospectively, as fatal PE can occur in patients with co-morbidities and not be recognized. – All fatality cases were explored reveal 8 unexpected death in the high-risk group (8/35, 22.8%) and 4 unexpected deaths in the low-risk group (4/17, 22.5%). This indicates no difference between the high and low risk groups in the incidence of unexpected death. The paragraph has been changed accordingly.
2. Representativeness of medical inpatients - The authors could provide more characteristics of the participants, such as the individual components of the Padua score. Some characteristics appear different from the Padua or ESTIMATE cohorts the authors cite, such as the low proportion of cancer (4-7%, vs. 20-27%). Overall, I would like to be convinced that included participants were acutely ill medical inpatients, and not rehabilitation or more geriatric inpatients. A sampling-weighted mortality estimated would also be interesting here. – Data obtained from **four** internal medicine departments. Geriatric patients were included since they are hospitalizing in internal medicine wards in our medical center. Rehabilitation patients were not included, they hospitalize in different ward. We do not have oncology ward so patients with VTE due to malignancy are hospitalizing in internal medicine wards. The average age was 75y. The proportion of cancer patients in our study, in our opinion, reflect the reality better than cited reports.
3. Definition of thromboprophylaxis - Could the authors provide the study operational definition for thromboprophylaxis? Was there a minimum duration, was it for specific drugs or doses? Thromboprophylaxis was defined as once daily treatment with an Enoxaparin, a low molecular weight heparin, at a dose of 0.5-1 mg/kg throughout the hospitalization for VTE prevention. This sentence was added to the method paragraph.
4. Calculation of the Padua score - The authors provide little information on how the score was calculated. Given that its evaluation is part of the inclusion criteria, was it prospectively calculated and entered in the electronic medical chart by in-charge hospital-physicians? If it was retrospectively calculated, there is a potential for misclassification that goes beyond of the immobility item and this should be discussed. What definition of immobility did the authors use? The Padua score was calculated prospectively and entered in the electronic medical chart by the attending hospital-physicians upon patients arrival to the ward. This sentence was added to the method paragraph.
5. Choice of the Padua score - Why was the Padua score selected for this analysis? Other scores are valid and more simple to implement, such as the Improve score (Spyropoulos, Chest 2011) or the simplified Geneva score (Blondon, JTH 2020). – We agree that all predictive models should be re-asses geographically for cost-benefit, efficiency and safety. We analyzed the Padua model since clalit health service, as with other HMO worldwide, proclaimed this model as quality measure for internal wards

Minor comments

1. Did none of the low-risk Padua score inpatients receive thromboprophylaxis? – Correct
2. Was extended thromboprophylaxis used in some participants? – Thromboprophylaxis defined as VTE treatment throughout the hospitalization and not further.
3. Why did the authors choose a time period in 2014, for a 2020 study? – It was the second year that all of the medical records were computerized, and the Padua score was applied.
4. I do not understand the second sample size calculation “In order to show that in the group with the low Padua score the VTE incidence is 1%, we would need to recruit 644 patients (at a 1:3 ratio, i.e. 161 patients with a high Padua score and 474 with a low Padua score), in order to achieve power 80% and alpha of 5% (two-tailed test).” Did the author forget to describe the comparison arm here? Comparison arm was done in the first sentences of the paragraph comparing the PEP in treated vs non-treated groups. “We estimated a VTE risk of 5% in our patients with PPS ≥4 points. In order to show that in the group that received thromboprophylaxis, the incidence of VTE decreases by approximately 80%, 568 patients were enrolled (284 in each group) in order to achieve power of 80% and alpha of 5% (two tailed test)”. The second analyses were comparing the High vs Low Risk group.
5. Were the 474 and 284 randomly sampled high-risk Padua score without thromboprophylaxis independent, or are there same individuals in both groups? Same individuals in both groups
6. I don’t understand why the assessment of mobility by the attending physician would change the observed incidence of VTE, given that most participants did not receive thromboprophylaxis anyway. – Mobility assessment would contribute 3 points to the Padua score. Incorrect filling of this criteria will result in placing the patient to the wrong risk group which then influence whether the patient will receive or not receive thromboprophylaxis. This may affect the incidence of the primary end points. Therefore, we explained it in the limitation paragraph.
7. Table 1: past VTE instead of past deep VTE – Corrected
8. Table 2 is improperly labeled as table 1. – Corrected

Reviewer #2:

1. Could a difference with regard to the underlying disease being responsible for the difference within the studies; eg in this analysis there is a really small number of renal disease patients, hematooncological patients. – The data in the study were randomly taken from four internal wards and included geriatric patients. The prevalence of co-morbidities among our patients did not differ when we compared it to other periods (we asses it follow reviewer comment) and we believe it faithfully represent our real population. Our patients may differ from other population worldwide, but this is exactly the message we want to convey from our research. Prior implement comprehensive and routinely treatment strategy for all hospitalized patients, the real incidence of VTE should be re-asses based on specific population or geographical areas. Previous studies have demonstrated a marked difference in the incidence of VTE events, some of which are probably explained by differences in the patient population.

Sincerely,

Dr. Koren Ofir
